# Analysis of the effect of cerium on the formation of non-metallic inclusions in low-carbon steel

**DOI:** 10.1038/s41598-023-34761-0

**Published:** 2023-05-22

**Authors:** Michał Szucki, Dorota Kalisz, Sergii Gerasin, Natalia Maria Mrówka, Jerzy Iwanciw, Sergey Semiryagin

**Affiliations:** 1 Gießerei-Institut der TU Bergakademie Freiberg, Freiberg, Germany; 2 AGH-University of Science and Technology in Krakow, Krakow, Poland; 3 Research and Design institute «Dneproenergostal» in Zaporozhye, Zaporozhye, Ukraine

**Keywords:** Engineering, Materials science

## Abstract

The research focused on the influence of cerium on the chemical composition and morphology of non-metallic inclusions in pre-oxidised steel to which Al, Ca, and Ce was added in different amount and order. Calculations were carried out using our own computer program. The simulation results obtained according to two calculation models helped identify precipitates from the Ce–O–S system. The possibility of CeN formation was also identified. The trace amounts of these inclusions were also found in the results. Consideration of the physicochemical phenomena at the boundary, as well as the interfacial partitioning and the sulfur partition coefficient, influences the favourable chemical composition of the inclusions, limiting it mainly to compounds from the Al_2_O_3_, Ce_2_O_3,_ and CaS systems. It was found that the addition of Ce before Ca causes the elimination of MnS precipitates and Ca-containing inclusions in the steel.

## Introduction

Problems related to the formation of non-metallic inclusions in steels are one of the leading research topics in metallurgy, as their impact on steel quality and properties is significant. The growing demand for high-quality steel is forcing manufacturers to introduce and continuously develop new materials to meet the expectations of customers. This is done by eliminating unfavorable non-metallic inclusions, controlled separation of desired particles, and modification of existing precipitates^1–3^. Rare earth metals known as lanthanides have special properties in this regard. These elements, due to their high electronegativity, are chemically very active in liquid steel, exhibiting high chemical affinity for oxygen, sulfur, and nitrogen, hence their action is much broader than, for example, aluminum, calcium, and titanium.

**Figure 1:**
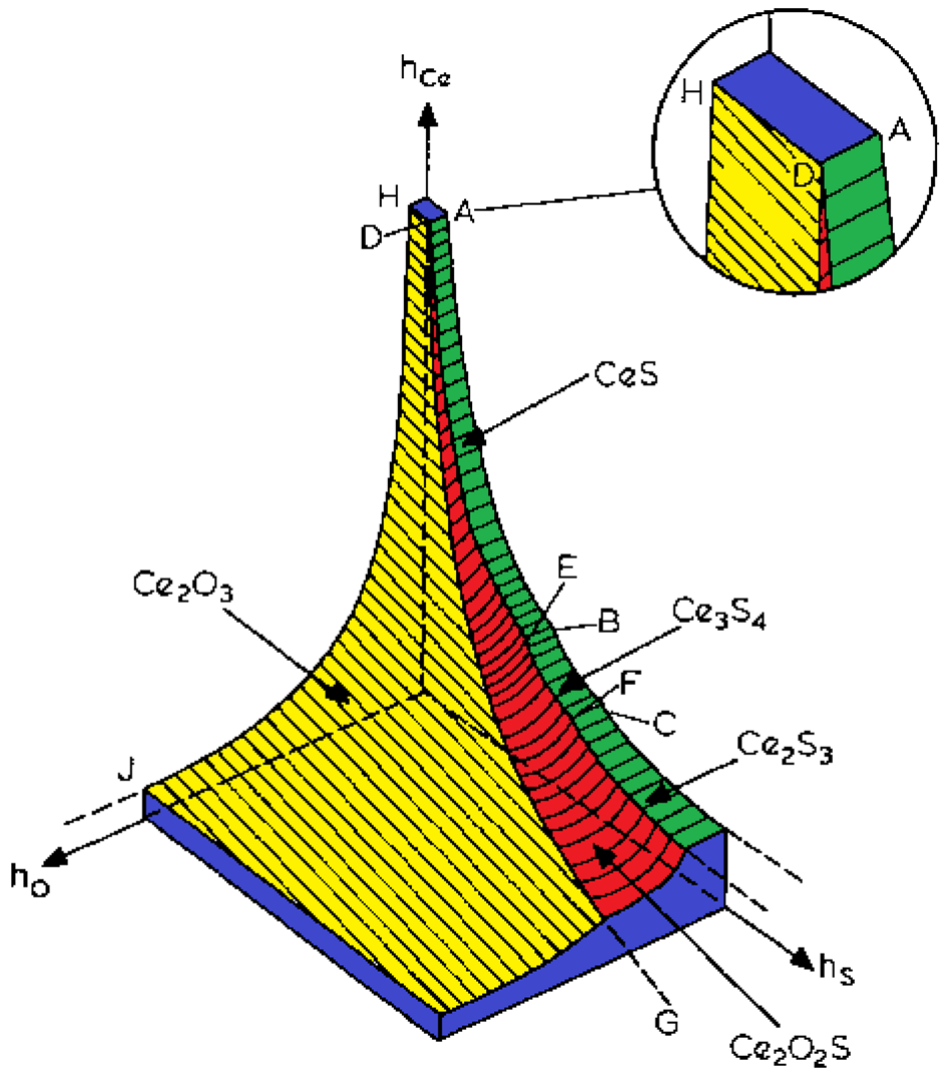
Phase system of Ce–O–S.

Cerium has a melting point of 799 °C and a density of 2989 kg/m^3^, while showing high solubility in liquid Feα (0.1–0.5%) and low solubility in Feγ (0.04%). Its main role is to deoxygenate, desulfurize and remove low melting point impurities by reacting with oxygen, sulfur and also arsenic, lead, tin and antimony. As a result of the reaction, it forms a number of compounds: oxides, oxosulfides, nitrides, and sulfides. Figure 1 shows the Ce–O–S equilibrium system, where the following phases are identified: Ce_2_O_3_, Ce_2_O_2_S, CeS, Ce_3_S_4_, and Ce_2_S_2_^4–8^.

The resulting compounds have high melting points, for example, Ce_2_O_3_ and CeO_2_ with melting points of 1690 °C and 1950 °C, respectively, and cerium-sulfur compounds: CeS (2450 °C), Ce_2_S_3_ (2149 °C), Ce_3_S_4_ (2080 ± 30 °C) and Ce_2_O_2_S (1950 °C)^9–11^.

The effect of cerium in steel is very complex because, in addition to its role as a deoxidizing and desulfurizing material, it interferes with the structure of inclusions and their chemical composition, morphology, and properties, consequently changing their plastic formability and corrosion resistance of steel. Mingya et al.^8^ presents studies on interfacial tension measurements of particles: SiO_2_, Al_2_O_3,_ and CeS in liquid steel. Low values of interfacial tension were found for CeS precipitates. This means that the presence of CeS in the liquid phase lowers the energy barrier for the formation of solid phase crystals several times. In high-sulfur steels, the authors observed that cerium sulfides act as crystallization centers, causing grain fragmentation and the formation of a zone of equiaxial crystals. On the other hand, in low-sulfur steels, the compound Ce_2_O_3_ was found, which can also act as a center for heterogeneous nucleation of other phases^12–16^. This was also confirmed by the research conducted by Adabavazeh et al.^17^, who found that Ce_2_O_3_ can act as nucleation sites for IAF. The study showed that the separation of cerium oxide, which has a high melting point, also affects the grain size in ultra-low carbon steels. It was found that the yield point significantly improved and the structure became fine-grained as a result of the increase in the number of nucleation centers of the solid phases.

Consequently, controlling the formation of desirable cerium-based inclusions favors higher strength and hardness of steel^12–16^. The fine-dispersed phase of Ce-containing precipitates can be used as centers for heterogeneous nucleation of sulfides, nitrides, or carbides, and the resulting cerium-containing precipitates inhibit grain growth and affect the formation of ferrite from austenite. As a result, they improve the mechanical properties, weldability, and fatigue strength, as well as corrosion resistance^18–20^.

In this work, authors investigated the behavior of cerium and its impact on the formation of non-metallic inclusions in steel using a computer program, the operation of which was described in previous work^21–27^.

## Materials and methods

The computer program WYK_STAL^25^ was used for making a real-time simulation of the refining process. The algorithm used in this program is presented in works^22,25^. The results of calculations carried out with the WYK_STAL program are comparable to those of industrial smelting. This is possible by matching such parameters as the size of the ladle, the method of mixing, and the mixing ratio. In the case of deoxygenation and desulfurization, hydrodynamic conditions are of particular importance. The transport of reactants consists of several stages. First, the reactant dissolves in the volume of metal and is distributed in it. Secondly, it diffuses through the boundary layer, so the concentration of reactants changes from average to equilibrium values corresponding to the equilibrium of a given reaction for the precipitate formation. For the purposes of the simulation, the mixing ratio has been determined on the basis of a thorough analysis of the smelting processes of 10 steels that are refined in a similar way. With the program, it is possible to control the dosage of alloying additives similarly to the real metallurgical process, where the process can take place continuously, in doses, or in a single dose. The program uses several calculation modules with such options as deoxidation, desulfurization, nitride formation, and verification of results. It is used to calculate the actual mass and chemical composition of the reacting phases at any point in the process, with the change in reactant concentration represented by a 1st-order kinetic equation. The WYK_STAL computer program has internal databases, physical and chemical parameters for steel and slag. The original version of the program was supplemented with the data given in Tables 1 and 2.

**Table 1:** The standard Gibbs energy for the given reactions is given in Table 1^17,28–30^.

No	Reactions	∆G_i_° /(J mol^–1^)
1	2 [Ce] + 3 [O] = Ce_2_O_3_ (s)	− 1,430,200 + 359 T
2	2 [Ce] + 2 [O] + [S] = Ce_2_O_2_ S(s)	− 1352,700 + 331 T
3	[Ce] + [S] = CeS (s)	− 394,428 + 121 T
4	3 [Ce] + 4 [S] = Ce_3_S_4_ (s)	− 1,494,441 + 439 T
5	2 [Ce] + Al_2_O_3_ (s) = 2 [Al] + Ce_2_O_3_ (s)	− 1,073,900 + 326 T
6	Ce_2_O_2_S (s) + [O] = Ce_2_O_3_ (s) + [S]	− 77,500 + 28 T
7	Ce_2_S_3_ (s) + [Ce] + [S] = Ce_3_S_4_ (s)	− 420,541 + 113 T
8	[Ce] + 5/2 [O] + 1/2 [Si] = 1/2 Ce_2_O_3_ (s) + SiO_2_ (s)	− 1,106,635 + 286.11 T
9	[Ce] + 3/2 [S] = 1/2 Ce_2_S_3_ (s)	− 536,420 + 163 86 T
10	[Ce] + [N] = CeN (s)	− 401,200 + 153.0 T

**Table 2:** The values of ${e}_{j}^{i}$ for liquid iron at 1,873 K^28,30–32^.

e $\frac{{\varvec{i}}}{{\varvec{j}}}$	C	O	S	P	Si	Mn	Al	N	Ce
O	− 0.45	− 0.20	− 0.133	0.07	− 0.131	− 0.021	− 3.90	0.057	− 12.1
S	0.11	− 0.27	− 0.028	0.029	0.063	− 0.026	0.035	0.01	− 1.88
Ce	0.351	− 106	− 8.225	1.77	−	0.13	− 2.67	−	0.0066
Mn	− 0.07	− 0.083	− 0.048	− 0.035	− 3.0	0	− 2.4	− 0.091	− 0.5
Al	0.091	− 6.6	0.03	0.033	0.0056	0.035	0.045	− 0.053	− 6.1
Si	0.18	− 0.23	0.056	0.11	0.11	0.002	0.058	0.090	− 7.7

The calculation of the formation of non-metallic inclusions in liquid steel can be performed in several variants depending on the type of model used and taking into account the properties of non-metallic inclusions. Variant 1 uses a model which assumes that the activity of the inclusion a = 1, in variant 2 the activity coefficient of metallic components f = 1, and lastly, variant 3 takes into account the metal-slag interfacial partition coefficient^25^. The set refining time was 30 min, the melt weight 140 Mg, the temperature of the liquid steel in the ladle 1670 °C, and the pressure 1 atm. The steel, with a chemical composition of 0.054% C, 0.05% Mn, 0.23% Si, 0.007% P, 0.005% N, and 0.01% S, was pre-oxidized hence the dissolved oxygen content of 0.01%. The slag in the ladle consisted of the following compounds: CaO—45%; Al_2_O_3_—2%; MgO—9%; MnO—5%; SiO_2_—12%; FeO—27%; this composition was supplemented by 100 kg of SiO_2_ in the 2nd minute of refining. Laboratory smelting was performed in a vacuum furnace and 2.2 g. Cerium was added to 1000 g of the pre-oxidized steel. Samples taken from the ingot were analyzed using a Tescan model VEGA3 W-REM scanning microscope equipped with a Bruker Nano XFlash 610 M EDS detector.

## Results and discussion

### Computer simulation

**Figure 2:**
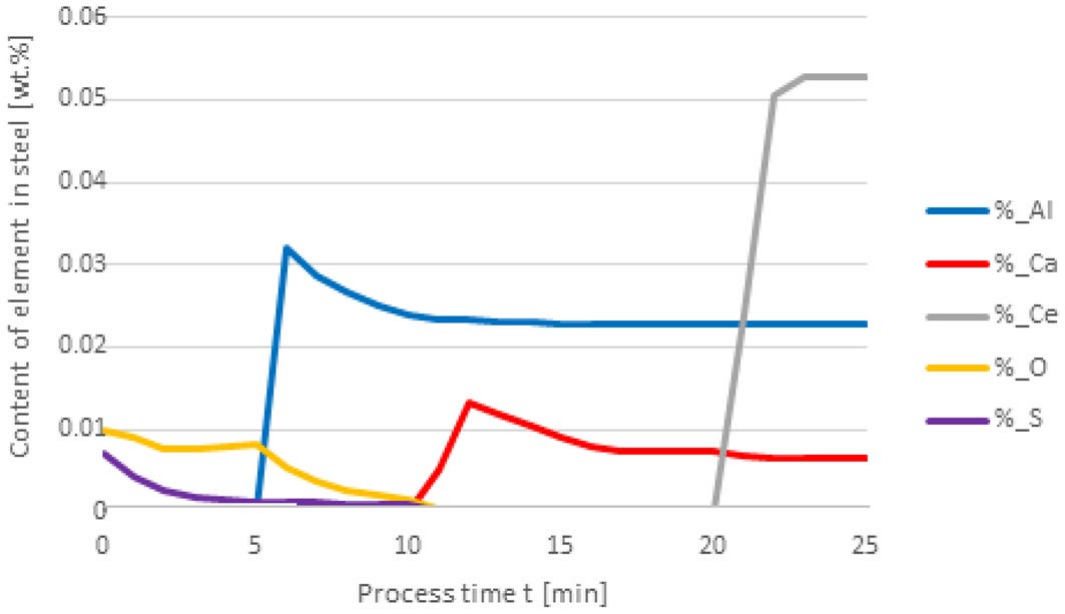
Change in the content of an element in liquid steel (wt%) depending on the process time, based on variant 1 calculations. Order of inserting alloying elements: 1st min—50 kg Al, 10th min—30 kg Ca, 20th min—75 kg Ce.

**Figure 3:**
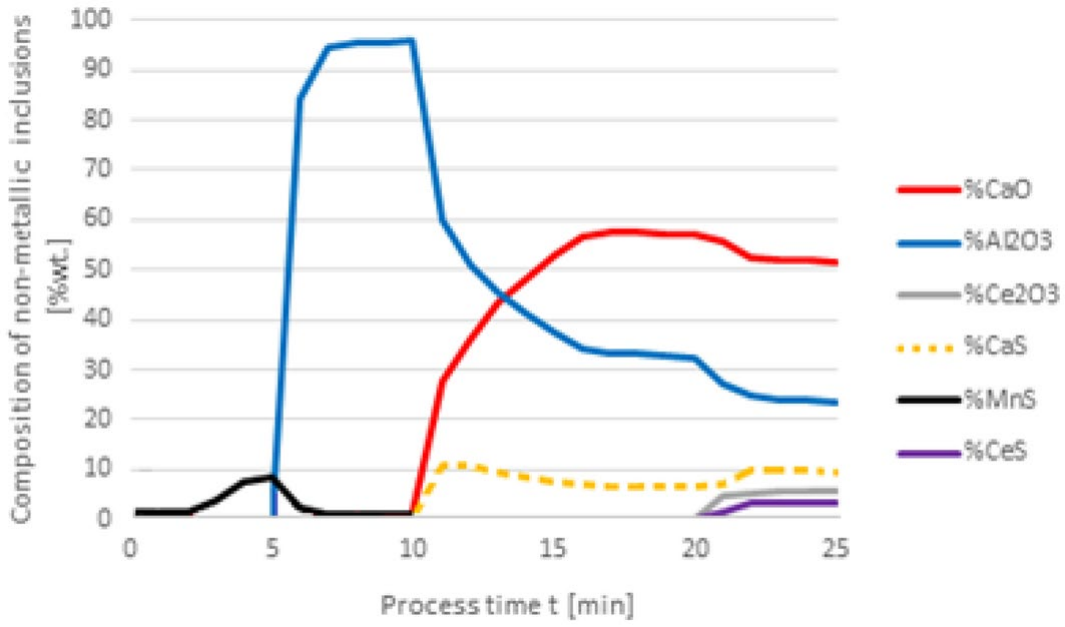
Change in the chemical composition of non-metallic inclusions (wt%) depending on the process time, based on variant 1 calculations. Order of inserting alloying elements: 1st min—50 kg Al, 10th min—30 kg Ca, 20th min—75 kg Ce.

Figures 2 and 3 show the results of calculating the change in the chemical composition of non-metallic inclusions calculated according to variant 1. At the first minute of the process, 50 kg of Al was added, then after 10 min, 30 kg of Ca was inserted, and finally, in the 20th minute, 75 kg of Ce was put in. The dosage of the components to the steel and the change in the chemical composition of the steel are shown in Fig. 2. The addition of aluminum caused the steel to deoxygenate to a level of 1 ppm, the introduction of calcium at the 10th minute of the process caused the sulfur content to drop to a level of 0.001% S, cerium, which was introduced at the 20th minute of refining, dissolved into the steel and its final content was at 0.0527%.

With this order of dosing additives, in the first phase of the process, Al_2_O_3_ inclusions are formed, the addition of calcium in the 10th minute of the process causes the formation of CaO and CaS precipitates. Calcium reacts with Al_2_O_3_ and as a result, CaO is formed. From the course of the line of change in sulfur content (Fig. 2), it is clear that it was consumed for the formation of CaS. The addition of cerium in the 20th minute of the process changes the percentage in the inclusions. Cerium has a higher chemical affinity for oxygen than aluminum, so it will modify Al_2_O_3_ inclusions, and CeAlO_3_-type inclusions may form as a consequence.

Then the dosage of the additives is changed in the following order: in 1st minute 50 kg Al in the 10th minute 75 kg Ce in the 20th minute 20 kg Ca. Then simulations, according to the variant 1 is performed. The addition of aluminum in the 1st minute of refining leads to the formation of Al2O3, and as a result of the reaction with cerium added in the 10th minute, its content changed according to reaction 5 (Table 1). As a consequence, the aluminum content in the steel is 0.013%. 10 min after the addition of cerium, the oxygen and sulfur content decreases.

**Figure 4:**
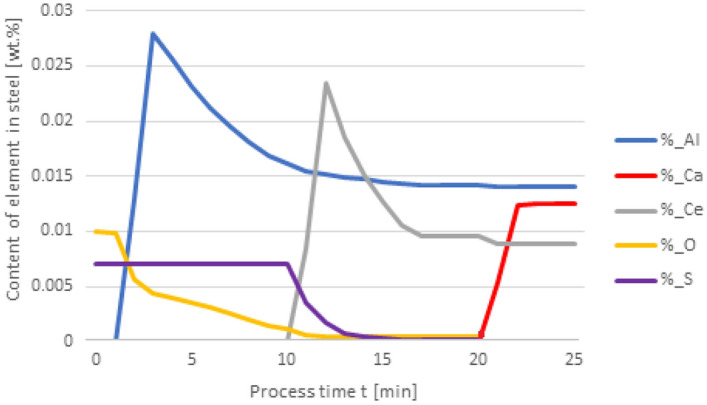
Change in the content of an element in liquid steel (wt%) depending on the process time, based on variant 1 calculations. Order of inserting alloying elements: 1st min—50 kg Al, 10th min—75 kg Ce, 20th min—20 kg Ca.

**Figure 5:**
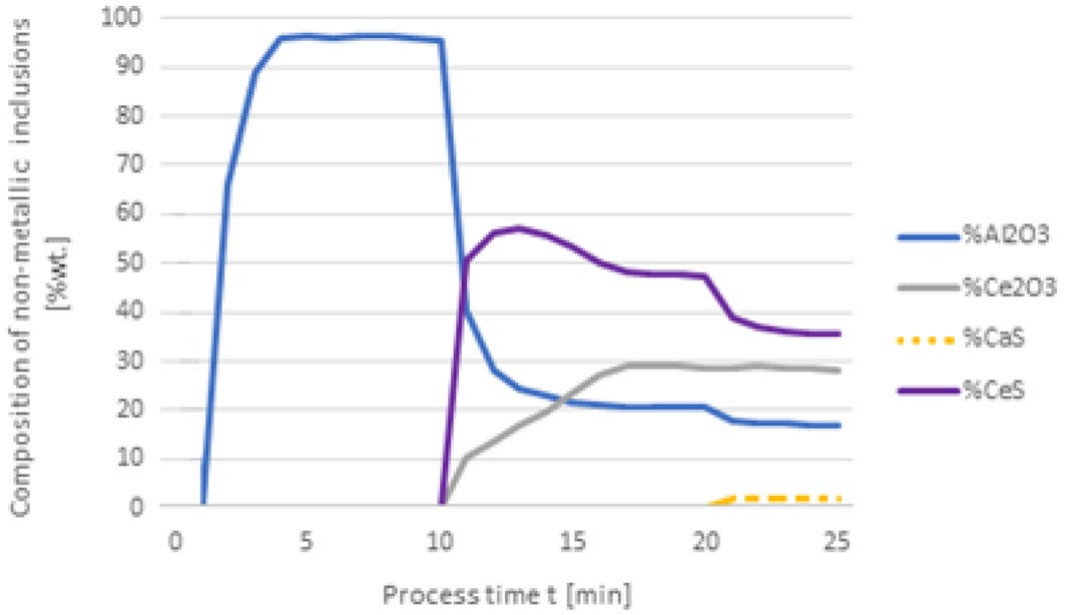
Change in the chemical composition of non-metallic inclusions (wt%) depending on the process time, based on variant 1 calculations. Order of inserting alloying elements: 1st min—50 kg Al, 10th min—75 kg Ce, 20th min—20 kg Ca.

Changing the order in which the additives are dosed results in an increased expenditure of cerium consumed during the formation of non-metallic inclusions. The results shown in Fig. 4 are interesting as the Ca addition at 20 min of the process results in its dissolution in steel and no inclusions are formed. It is also significant that the final content of cerium in steel is 0.05%, while for variant 1 in Fig. 2, it totals to 0.009%. The loss of cerium is caused by the formation of cerium sulfide and oxide inclusions, accompanied by a simultaneous increase of calcium in steel. In Fig. 5, the steel after refining is dominated by CeS inclusions, while in Fig. 3 mainly CaO and Al_2_O_3_ are observed. Unlike in Fig. 3, no MnS inclusions are present here.

**Figure 6:**
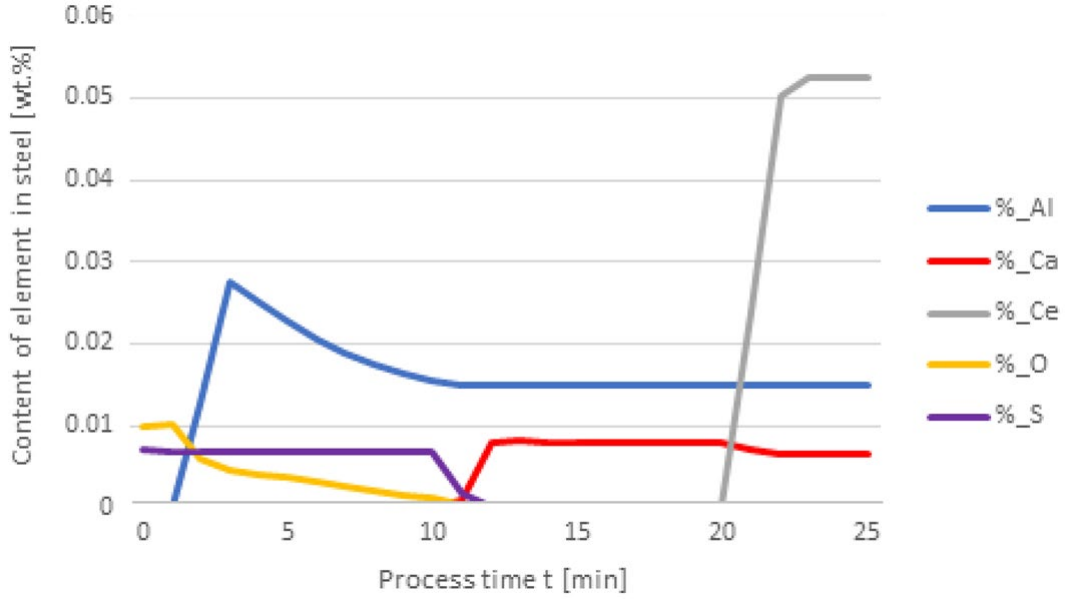
Change in the content of an element in liquid steel (wt%) depending on the process time, based on variant 3 calculations. Order of inserting alloying elements: 1st min—50 kg Al, 10th min—30 kg Ca, 20th min—75 kg Ce.

**Figure 7:**
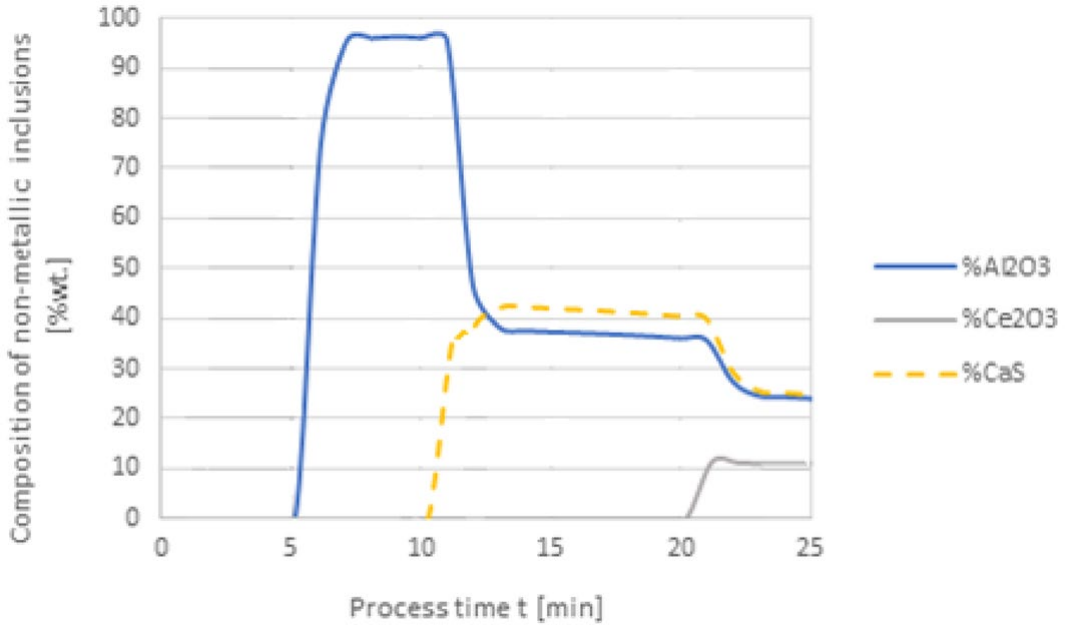
Change in the chemical composition of non-metallic inclusions (wt%) depending on the process time, based on variant 3 calculations. Order of inserting alloying elements: 1st min—50 kg Al, 10th min—30 kg Ca, 20th min—75 kg Ce.

**Figure 8:**
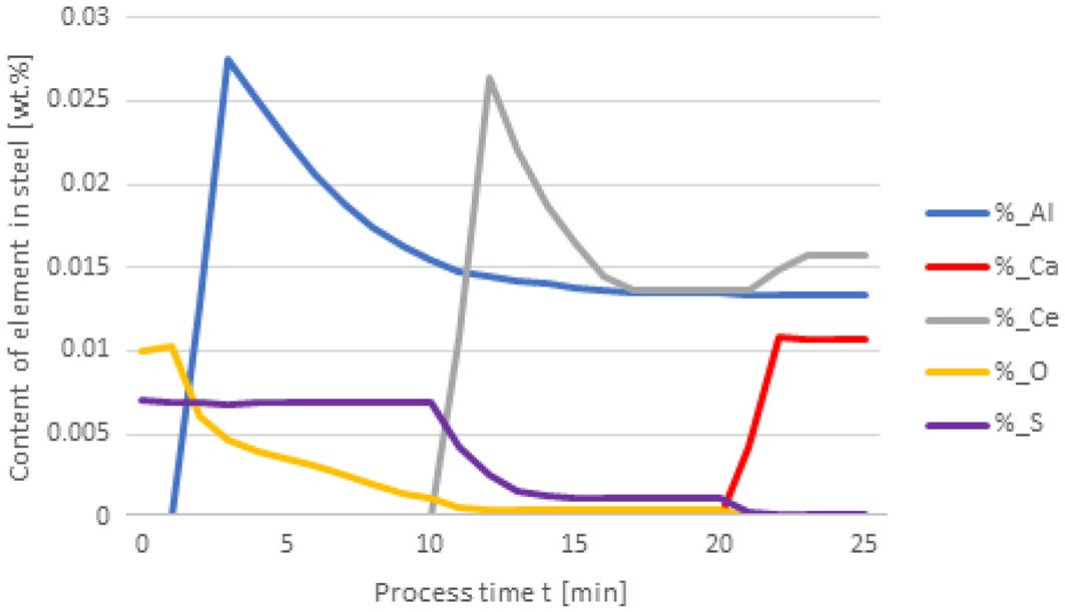
Change in the content of an element in liquid steel (wt%) depending on the process time, based on variant 3 calculations. Order of inserting alloying elements: 1st min—50 kg Al, 10th min—75 kg Ce, 20th min—20 kg Ca.

**Figure 9:**
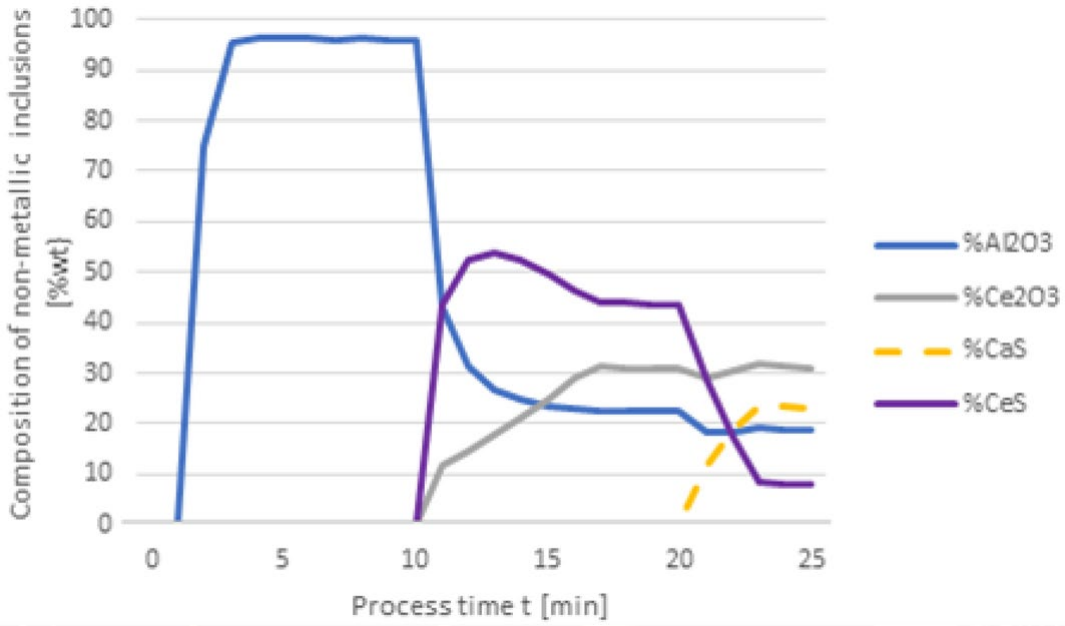
Change in the chemical composition of non-metallic inclusions (wt%) depending on the process time, based on variant 3 calculations. Order of inserting alloying elements: 1st min—50 kg Al, 10th min—75 kg Ce, 20th min—20 kg Ca.

A further series of calculations were performed for variant 3, taking into account the metal-slag interfacial partition coefficient. The results are shown in Figs. 6, 7, 8 and 9. As in the earlier calculations, the simulations were carried out for the same types of alloying additives dosing, i.e. 50 kg Al was added to steel at the 1st minute, 30 kg Ca at the 10th minute, and 75 kg Ce was introduced at the 20th minute. The simulation results are shown in Figs. 6 and 7.

The results of calculations for non-metallic inclusions according to variant 3 considerably differ from those for the model in variant 1. In this case, only Al_2_O_3_ and CaS inclusions are found in steel, the Ce_2_O_3_ precipitates are only formed after the introduction of cerium into deoxidised steel at the 20th minute of the process, there are no CeS precipitates, which were identified in calculations according to variant 1, and no MnS. The comparison of the results of individual elemental contents of the steel according to variants in Figs. 2 and 6 reveals a difference in the behaviour of sulfur; simulations for the model in variant 3 show that the sulfur content remains constant until the 10th minute of the process when calcium is introduced.

Further calculations were performed according to variant 3 where additives were introduced in the following order: 50 kg Al was added in the 1st minute, 75 kg Ce at the 10th minute, and 20 kg Ca at the 20th minute. The results are shown in Figs. 8 and 9.

When comparing the results of calculation models for variants in Figs. 4 and 8, attention is paid to the higher final Ce content in the steel. In contrast to the results given in Fig. 4, the introduction of calcium at the 20th minute of the process results in an increase in the Ce content of steel from 0.013% to slightly over 0.015%. This means that the loss of cerium is considerably lower than that resulting from calculations using variant 1.

In the case of non-metallic inclusions, the choice of calculation model also influences the percentage of individual inclusions in the steel. This is particularly true for CaS inclusions, which account for nearly their 20% presence in steel (Fig. 9), while for variant 1 (Fig. 5) their content is below 3%. The opposite is true for CeS, where the final content is below 10% (Fig. 9) and over 35% for the variant shown in Fig. 5.

There is therefore a direct correlation between sulfur and oxygen content as far as the formation of non-metallic inclusions in steel is concerned. As the steel was initially deoxidised and then further deoxidised with Al (Al_2_O_3_ was produced), the S/O ratio was high. This means that the addition of cerium at the 10th minute of refining results in the formation of cerium sulfide and can cause modification of the Al2O3 inclusions and the reactions (see reaction 3 and 5 in Table 1).

**Figure 10:**
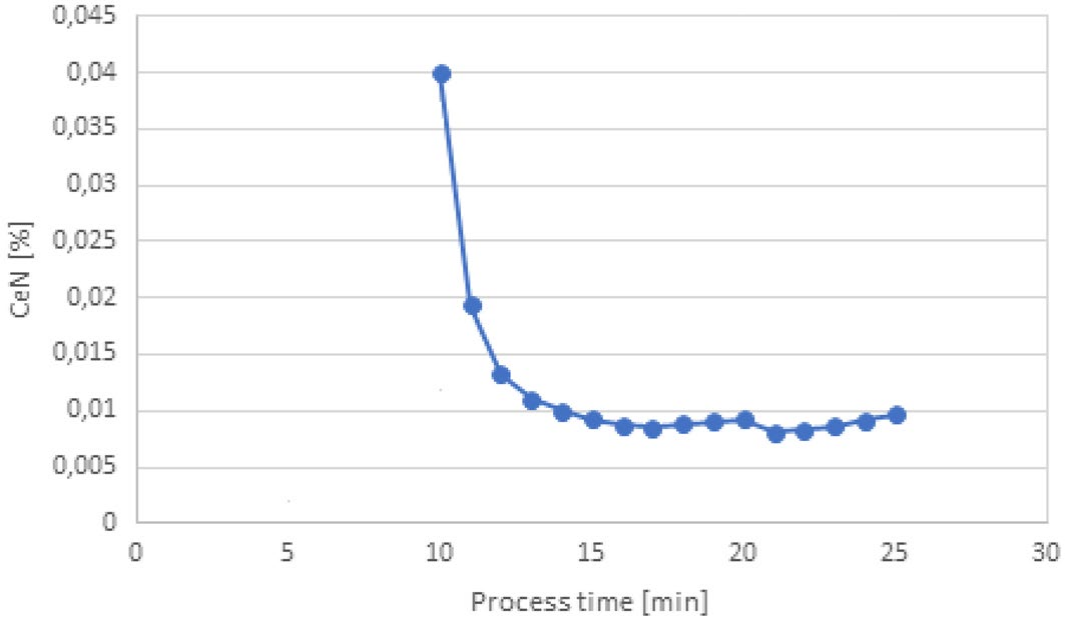
Change in the content of CeN (wt%) depending on the process time.

However, the formation of cerium oxysulfide is unlikely due to the low oxygen level of the pre-oxidised steel at the time of cerium dosage. Traces of CeN were identified in all analysed variants (Fig. 10).

### Microscopic analyses

**Figure 11:**
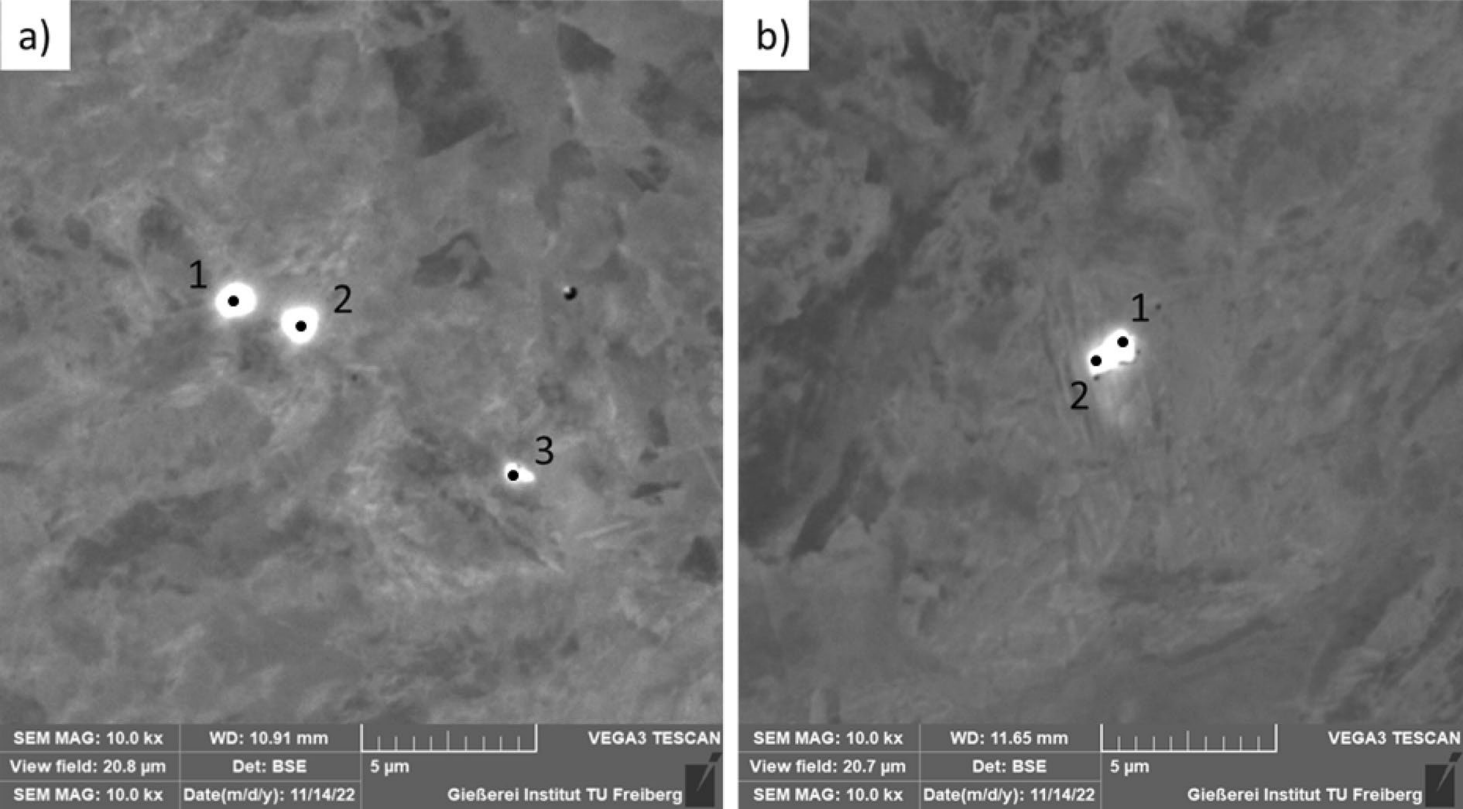
Microstructure of cerium-containing steel (**a**) sample I and (**b**) sample II made by scanning electron microscopy.

The smelting was carried out in an induction furnace with a 0.13 Pa vacuum. No alloying or deoxidising additives other than cerium were introduced during the process. The steel was then cast into a mould in an argon atmosphere, and samples were taken from various areas of the ingot. The essential examination by scanning electron microscopy was preceded by observation by optical microscopy. Numerous spherical, fine precipitates were identified in the samples (Fig. 11).

The presence of complex inclusions from the Fe–Ce–O–S system was identified in the tested samples. Analysis of the chemical composition showed that these phases contain the elements Ce, S, O (Table 3). This means that a Ce_2_O_2_S phase is formed, the presence of which is related to the presence of sulfur and oxygen deficiency in connection with deoxidation and vacuum melting (Supplementary Information 1).

**Table 3:** Result of spot X-ray microanalysis of the separation area points for samples I and II [mass %].

Point	C	N	O	Si	P	S	Mn	Fe	Ce
Sample I									
1	5.28	0.08	3.08	0.15	0.97	3.38	1.12	47.30	38.63
2	5.08	0.72	3.51	0.14	0.49	3.48	1.13	51.62	33.83
3	4.85	0.20	0.42	0.20	0.62	2.71	1.41	70.39	19.19
Sample II									
1	4.75	0.34	4.54	0.11	0.77	3.84	0.83	38.77	46.04
2	5.30	0.15	4.48	0.11	1.09	4.40	0.64	31.52	52.31

## Conclusion

This research examined the influence of cerium on the chemical composition and morphology of non-metallic inclusions in pre-oxidised steel. The results can be summarised as follows:

1. The *feeding of cerium at the final refining stage, after the addition of calcium, results in a reduction in its consumption for the formation of non-metallic inclusions, which are mainly CaO, CaS, and Al_2_O_3_ precipitates.
2. The addition of cerium before calcium leads to the loss of cerium mainly for the formation of Ce and Al precipitates. Small amounts of CaS are also formed with oxygen and sulfur.
3. The inclusion of the sulfur partition coefficient in the calculation affects the chemical composition of the non-metallic inclusions in the following way: when cerium is fed through the limestone, precipitates are formed: CeS, CaS, and Ce_2_O_3_ as well as Al_2_O_3_, with Ce–O–S inclusions accounting for about 40% after refining. In contrast, dosing calcium prior to cerium eliminates the formation of CeS.
4. It was concluded that CeN formation is possible for all analysed variants.
5. The analysis of the samples taken from the ingot confirmed the formation of fine, spherical precipitates and inclusions composed of the Ce–O–S system. The chemical composition of these inclusions suggests the formation of Ce_2_O_2_S compounds.

## Supplementary Information

Supplementary Information 1.

Supplementary Information 2.
