# Supplementary material for: Analysis of the effect of cerium on the formation of non-metallic inclusions in low-carbon steel
Source: Sci Rep. 2023 May 22;13:8294. doi: 10.1038/s41598-023-34761-0 (PMC10203100; doi:10.1038/s41598-023-34761-0)
Supplement: Supplementary file 1 — Supplementary Information 1. [file 41598_2023_34761_MOESM1_ESM.pdf]

Bericht REM

Gießerei Institut / TU Bergakademie Freiberg

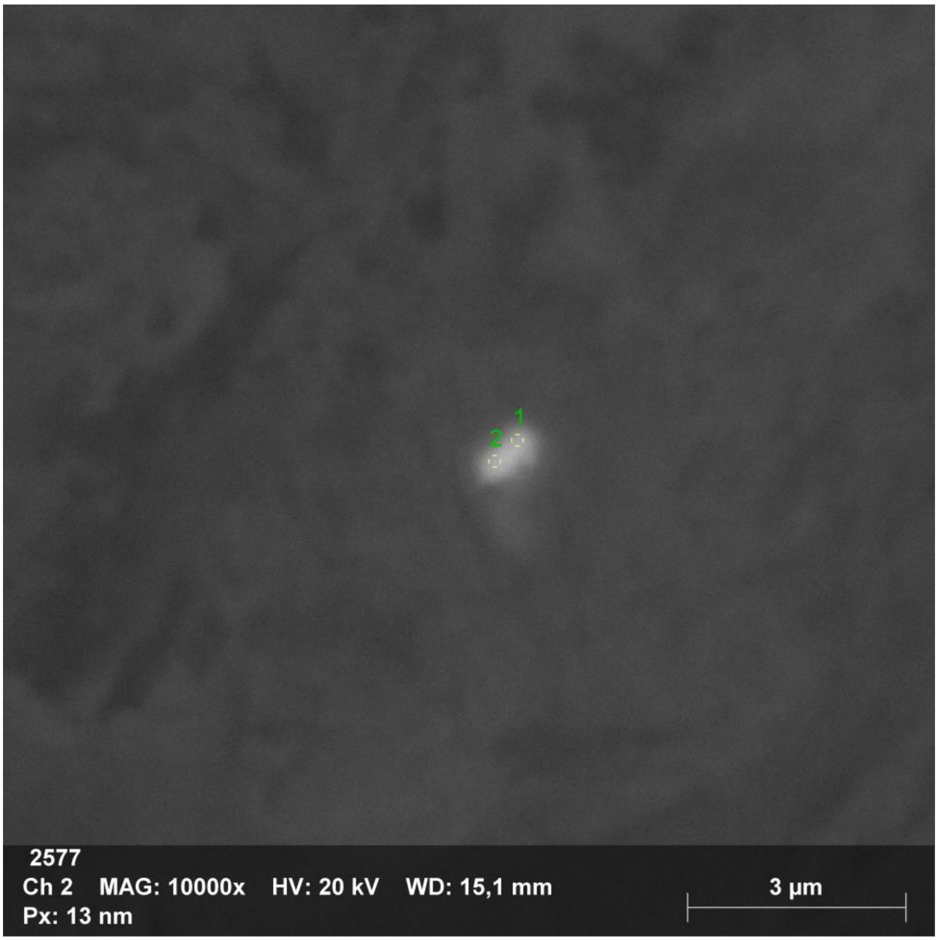

Normierte Massekonzentration [%]

Spectrum	C	N	O	Si	P	S	Mn	Fe	Ce
1	4,75	0,34	4,45	0,11	0,77	3,84	0,83	38,77	46,06
2	5,30	0,15	4,48	0,11	1,09	4,40	0,64	31,52	52,31
Mean	**5,03**	**0,25**	**4,51**	**0,11**	**0,93**	**4,12**	**0,73**	**35,14**	**49,18**
Sigma	**0,38**	**0,13**	**0,04**	**0,00**	**0,23**	**0,40**	**0,14**	**5,13**	**4,42**
SigmaMean	**0,27**	**0,09**	**0,03**	**0,00**	**0,16**	**0,28**	**0,10**	**3,63**	**3,13**

Bericht REM

Gießerei Institut / TU Bergakademie Freiberg

Stöchiometrische Konzentration [%]

Spectrum	C	N	O	Si	P	S	Mn	Fe	Ce
1	20,95	1,28	15,00	0,21	1,31	6,33	0,80	36,73	17,39
2	23,74	0,58	15,09	0,20	1,90	7,39	0,62	30,38	20,10
Mean	**22,34**	**0,93**	**15,04**	**0,21**	**1,61**	**6,86**	**0,71**	**33,56**	**18,74**
Sigma	**1,97**	**0,50**	**0,06**	**0,00**	**0,42**	**0,74**	**0,12**	**4,49**	**1,91**
SigmaMean	**1,40**	**0,35**	**0,04**	**0,00**	**0,30**	**0,53**	**0,09**	**3,17**	**1,35**

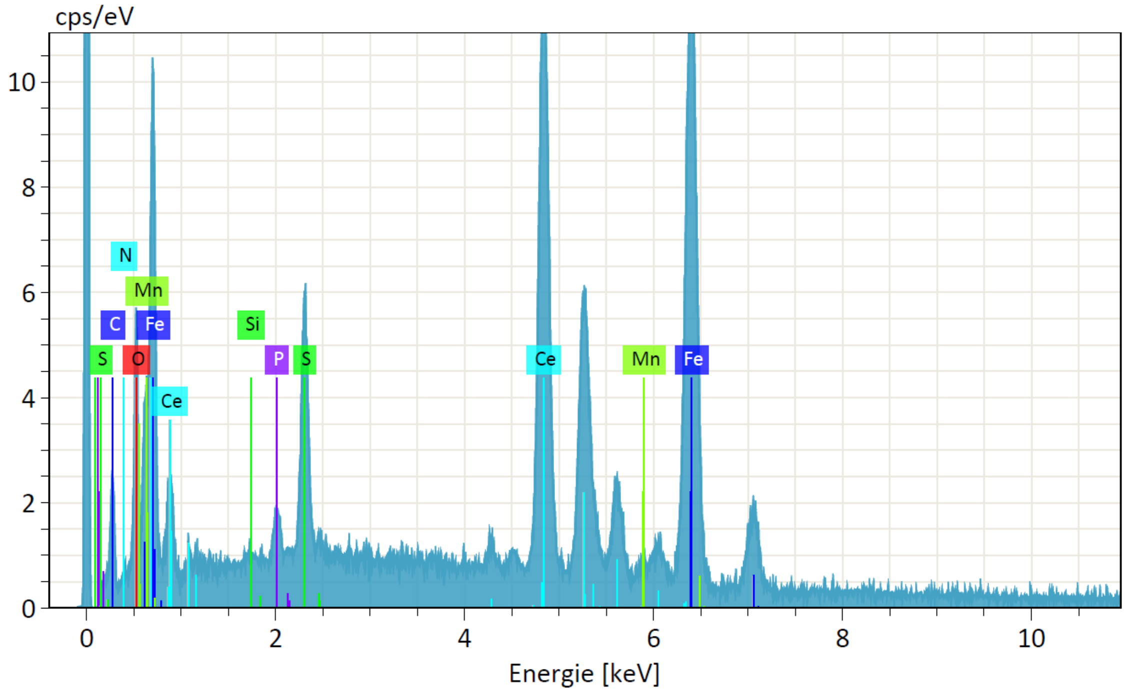

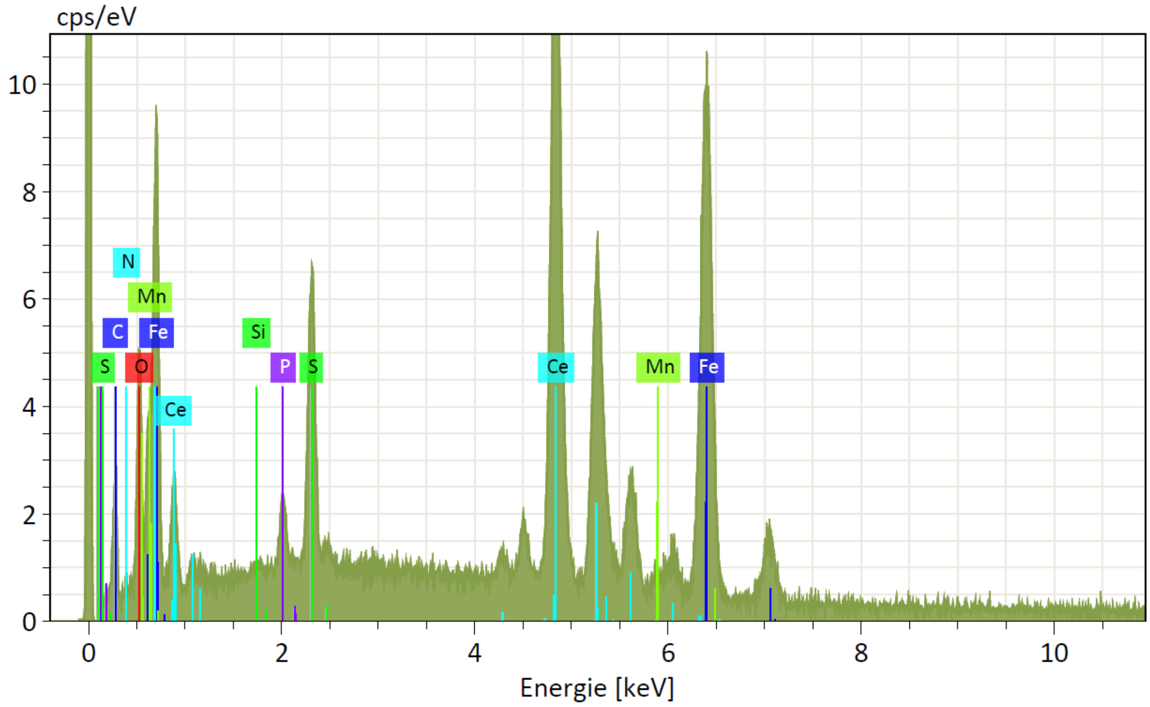
cps/eV
10
8
6
4
2
0
N
Mn
C
Fe
S
O
Ce
Si
P
S
Ce
Mn
Fe
0
2
4
6
8
10
Energie [keV]
