# Supplementary material for: Analysis of the effect of cerium on the formation of non-metallic inclusions in low-carbon steel
Source: Sci Rep. 2023 May 22;13:8294. doi: 10.1038/s41598-023-34761-0 (PMC10203100; doi:10.1038/s41598-023-34761-0)
Supplement: Supplementary file 2 — Supplementary Information 2. [file 41598_2023_34761_MOESM2_ESM.pdf]

Bericht REM

Gießerei Institut / TU Bergakademie Freiberg

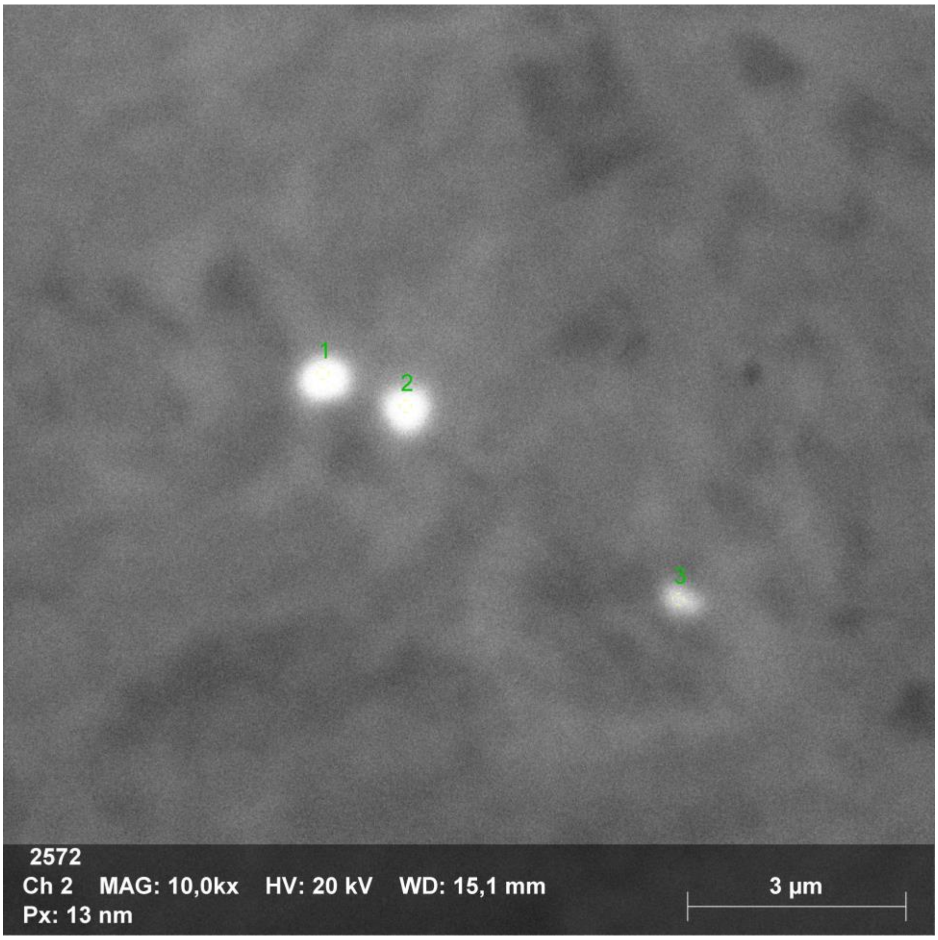

Normierte Massekonzentration [%]

Spectrum	C	N	O	Si	P	S	Mn	Fe	Ce
1	5,28	0,08	3,08	0,15	0,97	3,38	1,12	47,30	38,63
2	5,08	0,72	3,51	0,14	0,49	3,48	1,13	51,62	33,83
3	4,85	0,20	0,42	0,20	0,62	2,71	1,41	70,39	19,19
Mean	**5,07**	**0,33**	**2,34**	**0,16**	**0,69**	**3,19**	**1,22**	**56,44**	**30,55**
Sigma	**0,22**	**0,34**	**1,68**	**0,03**	**0,25**	**0,42**	**0,16**	**12,28**	**10,12**
SigmaMean	**0,12**	**0,20**	**0,97**	**0,02**	**0,14**	**0,24**	**0,09**	**7,09**	**5,85**

Bericht REM

Gießerei Institut / TU Bergakademie Freiberg

Stöchiometrische Konzentration [%]

Spectrum	C	N	O	Si	P	S	Mn	Fe	Ce
1	22,87	0,30	10,02	0,29	1,62	5,48	1,06	44,03	14,33
2	21,04	2,56	10,93	0,24	0,78	5,40	1,03	46,00	12,01
3	20,41	0,74	1,33	0,36	1,01	4,26	1,30	63,67	6,92
Mean	**21,44**	**1,20**	**7,42**	**0,30**	**1,14**	**5,05**	**1,13**	**51,24**	**11,09**
Sigma	**1,28**	**1,20**	**5,30**	**0,06**	**0,44**	**0,68**	**0,15**	**10,82**	**3,79**
SigmaMean	**0,74**	**0,69**	**3,06**	**0,04**	**0,25**	**0,39**	**0,09**	**6,24**	**2,19**

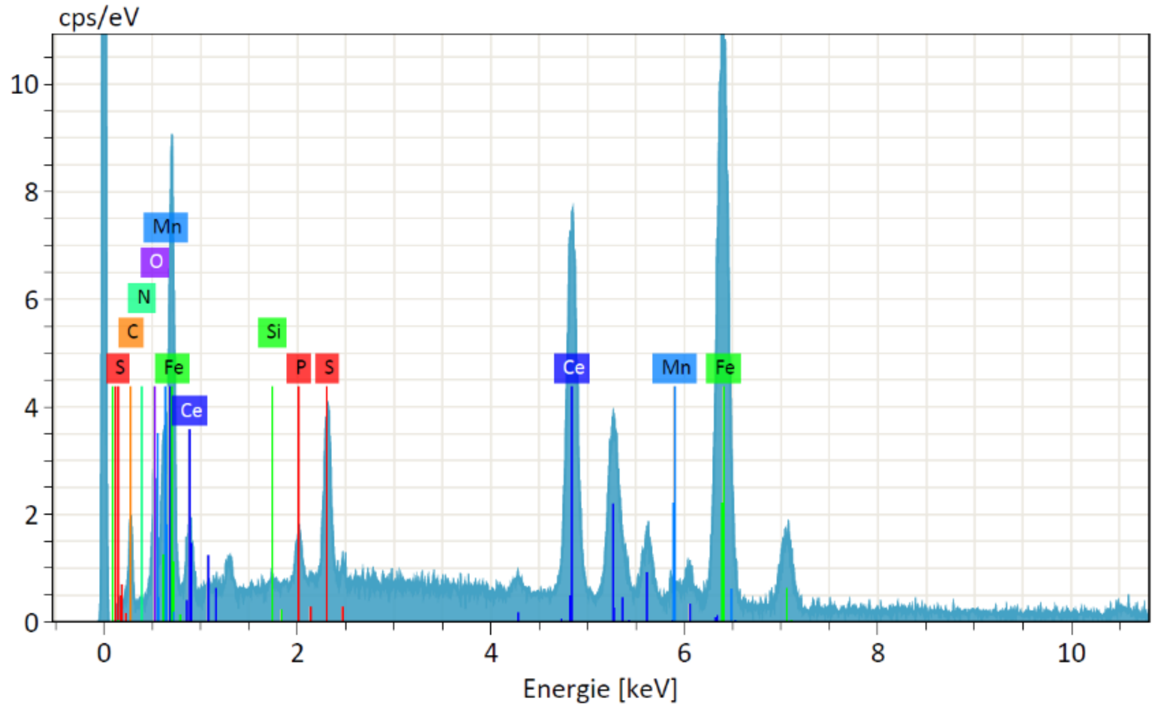

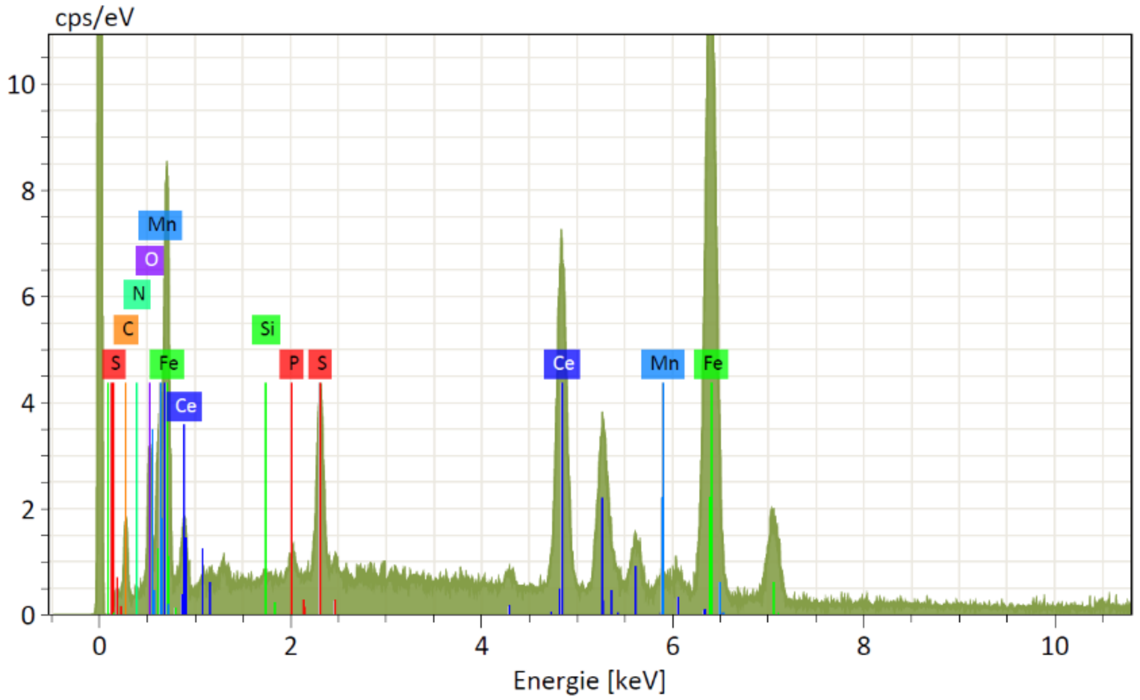
cps/eV
10
8
6
4
2
0
Mn
O
N
C
S
Fe
Ce
Si
P
S
Ce
Mn
Fe
0
2
4
6
8
10
Energie [keV]

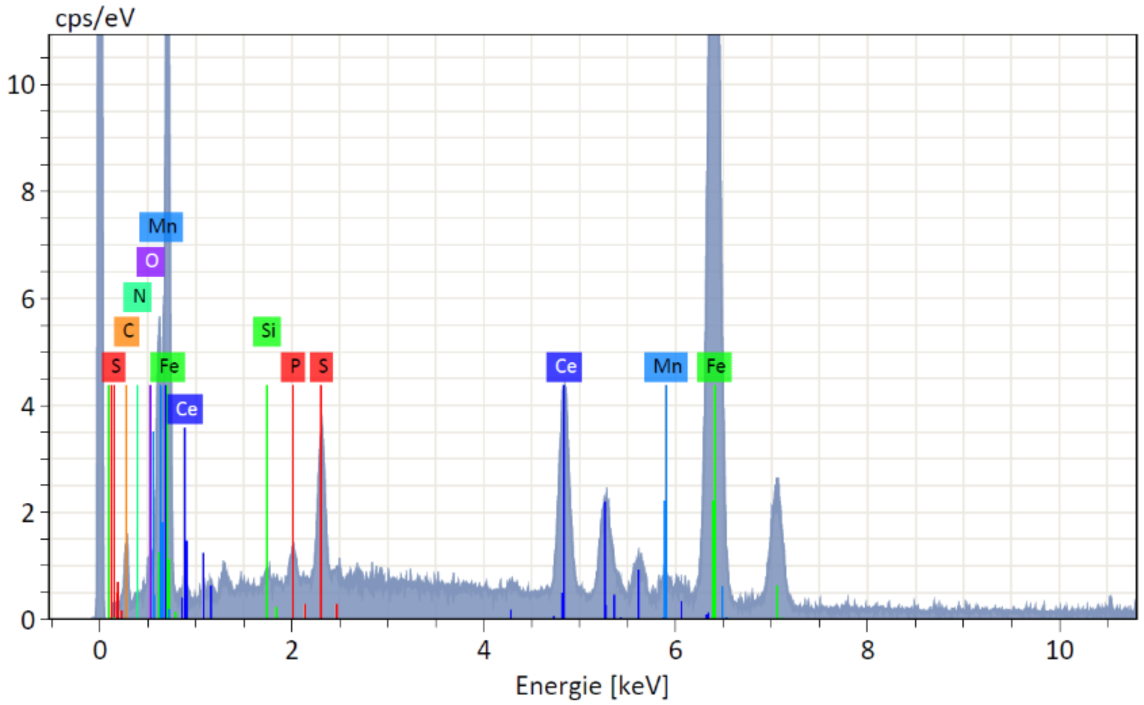
cps/eV
10
8
6
4
2
0
Mn
O
N
C
S
Fe
Ce
Si
P
S
Ce
Mn
Fe
0
2
4
6
8
10
Energie [keV]
